# Seroprevalence of hepatitis B, hepatitis C and HIV in ICU patients

**DOI:** 10.1186/cc14459

**Published:** 2015-03-16

**Authors:** H Bayir, I Yildiz, E Kocoglu, A Kurt, H Kocoglu

**Affiliations:** 1 Abant Izzet Baysal University, Medical School, Bolu, Turkey

## Introduction

Healthcare workers are at risk for infections caused by hepatitis B (HBV), hepatitis C (HCV) and human immunodeficiency (HIV) viruses that transmit via blood and body fluids. In the present study, it was aimed to investigate the seroprevalences of HBsAg, anti-HCV and anti-HIV in patients admitted to the ICU.

## Methods

HBsAg, anti-HCV and anti-HIV test results and demographical data of the patients admitted to the Reanimation ICU between January 2012 and December 2014 were evaluated retrospectively. HBsAg, anti-HCV and anti-HIV tests were assayed with a macro-ELISA method (Axsym-Abbott, Architect i2000; Abbott, USA). Statistical analysis was performed with the chi-square test.

## Results

The records of 462 patients admitted to our ICU were reviewed. The results of 36 patients could not be reached, so 426 patients were evaluated in the study. Among 426 patients, 169 (39.7%) were female and 257 (60.3%) were male. The mean age was 63.7 ± 18.7. HBsAg was positive in nine (2.1%) patients; all of these nine were male. Anti-HCV was positive in four (0.9%) patients; among these, three were male and one was female. Only one patient was positive for anti-HIV.

## Conclusion

In the present study, it was observed that the seroprevalences of HBsAg, anti-HCV and anti-HIV were not higher than in our city population. However, taking the safety precautions of the healthcare workers during surgical or invasive procedures such as catheterization, intubation or tracheostomy without any information about the serological test results of the patients will reduce the contamination of these agents.

## References

[B1] Erdem K Tas T Tekelioglu UY Bugra O Akkaya A Demirhan A The hepatitis B, hepatitis C and human immunodeficiency virus seroprevalence of cardiac surgery patients SDÜ Tip Fak Derg 2013 20 14 17

[B2] Tekin A Deveci O Seroprevalences of HBV, HCV and HIV among healthcare workers in a state hospital J Clin Exp Invest 2010 1 99 103

